# Systemic administration of valproic acid and zonisamide promotes differentiation of induced pluripotent stem cell–derived dopaminergic neurons

**DOI:** 10.3389/fncel.2013.00011

**Published:** 2013-02-15

**Authors:** Tatsuya Yoshikawa, Bumpei Samata, Aya Ogura, Susumu Miyamoto, Jun Takahashi

**Affiliations:** ^1^Department of Biological Repair, Institute for Frontier Medical Sciences, Kyoto UniversityKyoto, Japan; ^2^Department of Neurosurgery, Clinical Neuroscience, Kyoto University Graduate School of MedicineKyoto, Japan; ^3^Department of Neurosurgery, Mie University Graduate School of MedicineTsu, Japan; ^4^Department of Cell Growth and Differentiation, Center for iPS Cell Research and Application, Kyoto UniversityKyoto, Japan

**Keywords:** induced pluripotent stem cells, valproic acid, zonisamide, estradiol, transplantation, dopaminergic neurons

## Abstract

Cell replacement therapy using embryonic stem cells (ESCs) and induced pluripotent stem cells (iPSCs) is a promising strategy for the treatment of neurologic diseases such as Parkinson's disease (PD). However, a limiting factor for effective cell transplantation is the low survival rate of grafted cells, especially neurons. In this study, we modified the host environment and investigated whether the simultaneous administration of soluble factors can improve the survival and differentiation of murine iPSC-derived dopaminergic (DA) neurons in host brains. With the goal of applying this technology in clinical settings in the near future, we selected drugs that were already approved for clinical use. The drugs included two commonly used anti-convulsants, valproic acid (VPA) and zonisamide (ZNS), and estradiol (E2), also known as biologically active estrogen. Following neural induction of murine iPSCs, we collected neural progenitor cells (NPCs) by sorting PSA-NCAM^+^ cells, then treated the PSA-NCAM^+^ cells with drugs for 4 days. An immunofluorescence study revealed that 0.01 mM and 0.1 mM of VPA and 10 nM of E2 increased the percentage of tyrosine hydroxylase^+^ (TH: a DA neuron marker) cells *in vitro*. Furthermore, 0.1 mM of VPA increased the percentage of TH^+^ cells that simultaneously express the midbrain markers FOXA2 and NURR1. Next, in order to determine the effects of the drugs *in vivo*, the iPSC-derived NPCs were transplanted into the striata of intact SD rats. The animals received intraperitoneal injections of one of the drugs for 4 weeks, then were subjected to an immunofluorescence study. VPA administration (150 mg/kg/daily) increased the number of NeuN^+^ post-mitotic neurons and TH^+^ DA neurons in the grafts. Furthermore, VPA (150 mg/kg/daily) and ZNS (30 mg/kg/daily) increased the number of TH^+^FOXA2^+^ midbrain DA neurons. These results suggest that the systemic administration of VPA and ZNS may improve the efficiency of cell replacement therapy using iPSCs to treat PD.

## Introduction

Parkinson's disease (PD) is a progressive neurodegenerative disorder characterized by a loss of nigrostriatal dopaminergic (DA) neurons. In several clinical studies, the transplantation of fetal midbrain cells has successfully improved the motor symptoms of many PD patients, thus implying that cell replacement therapy is a promising strategy for the treatment of PD (Freed et al., 2001; Olanow et al., 2003; Mendez et al., 2005). More recently, pluripotent stem cells, especially induced pluripotent stem cells (iPSCs), have attracted much attention as a new source of donor cells due to their potential to supply a large quantity of DA neurons. However, iPSC-derived DA neurons and their precursors survive poorly in host brains (Hargus et al., 2010; Rhee et al., 2011). Furthermore, the procedure requires modification to improve the number as well as the quality of DA neurons in transplants to achieve the maximum efficacy of the therapy.

In the present study, we aimed to determine whether simultaneous administration of soluble factors can improve the survival and differentiation of murine iPSC-derived DA neurons in host brains. With the goal of applying this technology in clinical settings in the near future, we only used factors that were already approved for clinical use. Furthermore, candidate factors must cross the blood-brain barrier and diffuse into the brain parenchyma. Therefore, we selected two commonly used anti-convulsant drugs, valproic acid (VPA) and zonisamide (ZNS). We also selected estradiol (E2), also known as biologically active estrogen, an important female sex hormone that is widely used to treat patients suffering from ovarian deficiency symptoms, menopausal disorders, and osteoporosis.

ZNS has been shown to be neuroprotective in 6-OHDA- (Asanuma et al., 2010) and MPTP-lesioned mice (Yano et al., 2009; Sonsalla et al., 2010; Choudhury et al., 2011). Neuroprotective effects against MPTP toxicity are also observed with 17β-E2 (Dluzen et al., 1996, 2001; Miller et al., 1998; Callier et al., 2001; Ramirez et al., 2003) and VPA (Kidd and Schneider, 2010, 2011). Furthermore, these compounds are also advantageous in cell transplantation studies using neural progenitor cells (NPCs). A study by Abematsu et al. demonstrated remarkable improvement of the hind limb function in a mouse model of spinal cord injury after NPC transplantation with VPA treatment, which favors NPC differentiation toward neurons rather than glial cells (Abematsu et al., 2010). E2 increases the proportion of human NPC-derived DA neurons *in vitro* and *in vivo*, thus suggesting that it promotes DA differentiation and supports the survival of mature DA neurons (Kishi et al., 2005).

Based on these previous studies, we investigated whether VPA, ZNS, or E2 affects the differentiation of DA neurons derived from murine iPSCs *in vitro*. We then evaluated their effects *in vivo* by grafting the iPSC-derived NPCs into the striata of rats that received daily injections of one of the test compounds.

## Materials and methods

### Differentiation of dopaminergic neurons from murine iPS cells

A murine iPS line 440A-3 (a gift from Dr. Okita, Kyoto University Center for iPS Cell Research and Application, Kyoto, Japan) was used after 10–25 passages. Generated with a plasmid vector containing three genes, *Oct3/4, Klf4*, and *Sox2*, the 440A3 cells carried the green fluorescence protein (GFP) and the puromycin-resistance gene under the *Nanog* enhancer and promoter, which are only active when the cells are in an undifferentiated state (Okita et al., 2008). No integration of the exogene was reported.

**Figure 1:**
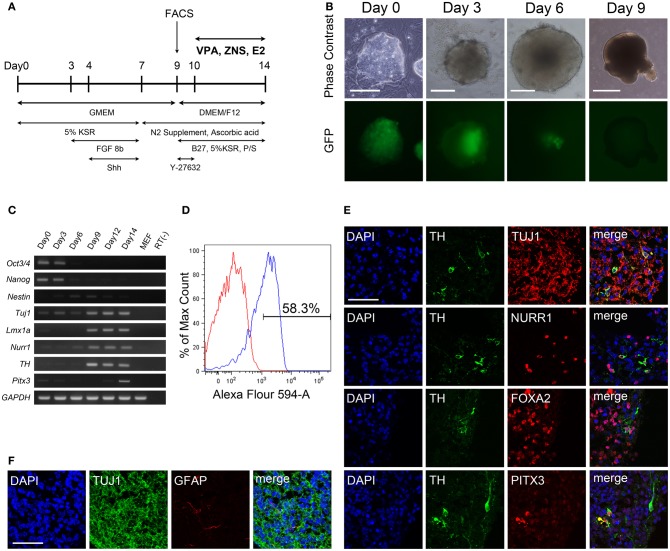
**Generation of dopaminergic neurons from murine iPSCs. (A)** Murine iPSCs (440A3) were induced to differentiate into DA neurons via the SFEBq method with the addition of various factors during 14 days of suspension culture. **(B)** Phase contrast images (upper) and fluorescent images for nanog-GFP (lower) of the suspension culture over time. Scale bars = 100 μm (day 0), 200 μm (days 3, 6), and 500 μm (day 9). **(C)** An RT-PCR analysis illustrating the temporal changes in the expressions of genes associated with pluripotency (Oct3/4, nanog), immature NPCs (nestin), post-mitotic neurons (Tuj1), and DA neurons (Lmx1a, Nurr1, TH, *Pitx3*). GAPDH was used as a control. **(D)** A flow cytometric profile of the population at day 9 labeled by the neural cell adhesion molecule PSA-NCAM. **(E,F)** Immunocytochemistry of a sliced aggregate fixed on day 14 indicating the presence of DA neurons **(E)** and astrocytes **(F)** in the culture. The scale bar applies to all pictures and represents 50 μm.

Undifferentiated cells were maintained on mitomycin C-treated murine embryonic fibroblast (MEF) feeder cells in DMEM (Wako) supplemented with 1% fetal calf serum, 5% knockout serum replacement (KSR; Invitrogen), 0.1 mM of non-essential amino acids, 1 mM of sodium pyruvate, 0.1 mM of 2-mercaptoethanol (2-ME; Invitrogen), 2000 U/ml of leukemia inhibitory factor (Invitrogen), and 1.5 μg/ml of puromycin (Takara) to eliminate differentiated cells. For neural induction of iPS cells, we used the serum-free culture of embryoid body-like aggregates (SFEB) method (Watanabe et al., 2005). Briefly, 440A3 cells were dissociated with 0.25% trypsin/1 mM EDTA and seeded onto 96-well low-adhesion plates (Lipidure-Coat Plate A-U96, NOF Corporation) at a density of 3000 cells/well to induce re-aggregation on day 0 in differentiation medium containing GMEM with 5% KSR, 0.1 mM of non-essential amino acids, 1 mM of sodium pyruvate, and 0.1 mM of 2-ME. During the differentiation period, various factors were added to induce the midbrain DA phenotype, as indicated in Figure 1A: 20 ng/ml of murine FGF-8b (R&D Systems) from days 3 to 7, 10 ng/ml of recombinant murine sonic hedgehog (C25II) N-terminus (R&D Systems) from days 4 to 7, 1% N-2 Supplement (Gibco) and 200 nM of ascorbic acid from day 7 onwards. KSR was withdrawn from the differentiation medium on day 7.

### Fluorescence-activated cell sorting (FACS)

On day 9, 440A3 cells were rinsed twice in PBS(–) and dissociated into single cells using a 5-min incubation with Accumax (Innovate Cell Technologies) at 37°C. The cells were collected with a FACS buffer consisting of PBS(–) with 2% FBS, 20 mM of D-glucose and 1% Penicillin/Streptomycin (P/S, Invitrogen), and mechanically dissociated into a single cell suspension by gentle pipetting. Subsequently, the cells were incubated with murine anti-PSA-NCAM antibodies (1:200, Millipore) for 30 min at 4°C and washed twice by centrifugation, followed by another 30-min incubation with the secondary antibody AlexaFluor 594 donkey anti-mouse IgG (1:400, Invitrogen). Dead cells and debris were excluded using 7-aminoactionomycin-D (7-AAD, BD Pharmingen) staining, and the viable cells were again suspended at a final concentration of 1 × 10^7^ cells/ml. Cell sorting was performed using a FACSAriaII cell sorter (Becton Dickinson) equipped with 488-nm argon and 633-nm Helium-Neon lasers, a 100-μm nozzle, and the FACSDiva software program. PSA-NCAM positivity was determined according to the negative control lacking the primary antibody.

### *In vitro* treatment of dopaminergic progenitors with test compounds

After cell sorting, the PSA-NCAM^+^ population was seeded onto 96-well plates at a density of 20,000 cells/well in DMEM/F12 medium (Wako) supplemented with 1% N-2 Supplement, 200 nM of ascorbic acid, 2% B27 Supplement (Invitrogen), 0.5 mM of L-glutamine, and 1% P/S to induce re-aggregation. The ROCK inhibitor Y-27632 (Wako) was used during the sorting procedure and the following overnight culture at 30 μM to prevent apoptosis (Koyanagi et al., 2008). On day 10, either VPA (Sigma), ZNS sodium salt (provided by Dainippon Sumitomo Pharma, Osaka, Japan), 17β E2 (Sigma), GDNF (R&D Systems), or PBS(–) was added to the culture for 4 days. VPA, ZNS, and E2 were each used at three different concentrations: 0.01 mM, 0.1 mM, and 1 mM for VPA, 1 μM, 10 μM, and 100 μM for ZNS, and 1 nM, 10 nM, and 100 nM for E2. GDNF was added at 20 mg/ml to provide a positive control. To antagonize the effects of VPA and E2, either an adenylate cyclase inhibitor 2,5-dideoxyadenosine (ddA, 100 μM; Santa Cruz Biotechnology) or an estrogen receptor antagonist ICI182780 (ICI, 2 μM; Wako) was added to the culture medium on day 10.

### Transplantation study

Ten-week-old male Sprague–Dawley rats (Shimizu Laboratory Supplies, Kyoto, Japan) were cared for and handled according to the Guidelines for Animal Experiments of Kyoto University. The animals were anesthetized and injected stereotactically with the donor cells in the bilateral striatum (from the bregma: A +1.0 mm; R or L +3.0 mm; V +4.5 mm). For each tract, two aggregations of the day 9 population containing 3.1 × 10^5^ cells on average were collected in 1 μl PBS(–) supplemented with 30 μM of Y-27632 and used for transplantation. Intraperitoneal injections of VPA (150 mg/kg/day), ZNS sodium salt, (30 mg/kg/day), E2 (80 μg/kg/day), or saline were administered 2 days in advance of the procedure and continued until the day of sacrifice. All animals also received a daily dose of 10 mg/kg of cyclosporine A (CsA, Wako) for immunosuppression. At 4 weeks post-transplantation, the animals were intracardially perfused with 4% paraformaldehyde under deep anesthesia. On the day of sacrifice, blood samples were collected from each animal 1 hour after the final injections of the test drugs or CsA. These samples were sent to SRL, Inc. (Tokyo, Japan) for measurement of the blood concentrations of these drugs.

### Reverse transcription-polymerase chain reaction (RT-PCR)

Total RNA was extracted using an RNeasy Plus Mini kit (Qiagen), and reverse transcribed using the Super Script III First-Strand Synthesis System (Invitrogen). PCRs were performed using Hot StarTaq DNA polymerase (Qiagen). For each primer, a control amplification reaction was performed without the addition of reverse transcriptase. MEF was used as the other negative control. The primer sequences and product sizes are shown in Table 1.

**Table 1:** **Summary of primers for RT-PCR**.

**Gene name**	**Forward primer**	**Reverse primer**	**Product size**
*Oct3/4*	CCTGGGCGTTCTCTTTGGAAAGG	GTAGGGAGGGCTTCGGGCACTT	263
*Nanog*	AGCAATGGTCTGATTCAGAAGGGCTC	AAATGCGCATGGCTTTCCCTAGTG	368
*Nestin*	GGCTTCTCTTGGCTTTCCTGACCC	GGGGGACATCCTGGGCTCTGAC	269
*Tuj1*	GGGCCAAGTTCTGGGAGGTCATC	GTCCAAAGGCGCCAGACCGA	198
*Lmx1a*	CCAGAACCAGCGAGCCAAGATGA	AGGCATCTGGGGTGGGGTGAG	238
*Nurr1*	GCGCTTAGCATACAGGTCCAACCC	CCTTGAGCCCGTGTCTCTCTGTGA	212
*TH*	TCCGGGCTTCTCTGACCAGGC	GCCAGTCCGTTCCTTCAAGAAGTGAG	280
*Pitx3*	GGGACGCACTAGACCTCCCTCCAT	AAGCCACCTTTGCACAGCTCC	420
*GAPDH*	CTCATGACCACAGTCCATGCCATCA	TCATCATACTTGGCAGGTTTCTCCAGG	251

### Immunofluorescence study

For the *in vitro* experiments, on day 14, drug-treated cell aggregates were fixed in 4% paraformaldehyde, frozen and cut into 10-μm-thick slices using a microtome for immunocytochemistry. Following the *in vivo* experiments, the brains were removed and re-fixed for two days in 4% paraformaldehyde, cryopreserved in 30% sucrose for 3 days, frozen, and cut into 40-μm-thick slices for immunohistochemistry. Frozen sections of the spheres and brains were permeabilized and blocked with 0.3% Triton X-100 and 2% donkey serum in PBS(–) for 1 h at room temperature, followed by overnight incubation with primary antibodies at 4°C. The primary antibodies used in this study included rabbit anti-tyrosine hydroxylase (1:400, TH;Millipore), mouse anti-TH (1:200, Millipore), sheep anti-TH (1:400, Millipore), mouse anti-Tubβ3 (1:1000, Tuj1; Covance), rat anti-NURR1 (1:1000, a gift from Dr. Ono, KAN Research Institute, Kobe, Japan), rabbit anti-Ki67 (1:1000, Novocastra: NCL-Ki67p), rabbit anti-Caspase3 (1:500, Santa Cruz Biotechnology), rat anti-M2M6 (1:50, Developmental Studies Hybridoma Bank), mouse anti-Nestin (1:500, Millipore), rabbit anti-Pitx3 (1:500, Chemicon), goat anti-HNF-3β (1:500, Foxa2; Santa Cruz Biotechnology), and mouse anti-NeuN (1:500, Chemicon). After three washes with 0.05% Tween-20 in PBS, the samples were incubated with Alexa Fluor-conjugated secondary antibodies at room temperature for 1 h. Following three additional washes, the samples were incubated with DAPI for nuclear staining and mounted using Permafluor (Dako). The immunoreactive cells were visualized with a confocal laser microscope (Fluoview FV1000D; Olympus, Tokyo, Japan). To determine the percentage of positive cells for each marker, labeled cells were manually counted for at least three independent experiments. The graft volume and number of Ki67^+^/Nestin^+^ cells were determined by identifying M2M6^+^ areas in every sixth 40-μm-thick section using the BZ-II Analyzer software program (Keyence) and totaling the volumes of all 240-μm-tall cylinders according to Cavalieri's principle. To estimate the number of immuno-reactive cells in each graft, the cells were manually counted in every sixth section, and the Abercrombie correction was applied (Abercrombie, 1946).

### Statistical analysis

The statistical analyses were performed using the GraphPad Prism software program Ver. 5.0 b (GraphPad Software). All quantitative data are presented as the mean value ± SD, and One-Way ANOVA and Newman–Keuls *post-hoc* tests were used. Differences were considered to be statistically significant for *P* < 0.05.

## Results

### Generation of DA neurons from murine iPSCs

Murine iPSCs (440A3) were induced to differentiate into DA neurons via the SFEB method with the addition of FGF 8 b and Shh, as described in Figure 1A. On day 0, dissociated iPSCs quickly re-aggregated in each well, and these cells proliferated continuously over 14 days in culture (Figure 1B). Along with the differentiation, the expression of Nanog-GFP gradually decreased until day 9, when it almost disappeared. The temporal changes in the gene expression profile shown in Figure 1C clearly illustrate the step-wise differentiation of the *Oct*3/4^+^ and *Nanog*^+^ pluripotent population on day 0 into *Nestin*^+^ immature NPCs around days 6–9 then into *Tuj*1^+^ neurons as they began to express the markers specific for DA subtype such as *Lmx1a, Nurr1*, and *TH*.

Although DA neurons were successfully generated in our suspension culture, undifferentiated iPSCs and non-neural cells possibly remained. In order to obtain a more homogenous population of NPCs, we used FACS to select for cells positive for PSA-NCAM, a cell adhesion molecule that is specifically expressed on the surface of neural cells (Bonfanti, 2006). On day 9, ~60% of the cells were positive for PSA-NCAM (Figure 1D). Following FACS, PSA-NCAM^+^ cells were made to re-aggregate and allowed to mature for another 5 days, then subjected to immunocytochemical studies on day 14. Immunofluorescent staining of the sliced aggregates revealed that most of the cells were TUJ1^+^ neurons, and among them were some mesencephalic DA neurons that simultaneously expressed TH, NURR1, FOXA2, and PITX3 (Figure 1E). Only a few (<0.1% of the total number of cells) cells expressed GFAP, a marker for astrocytes (Figure 1F).

### VPA and E2 increased dopaminergic neurons *in vitro*

**Figure 2:**
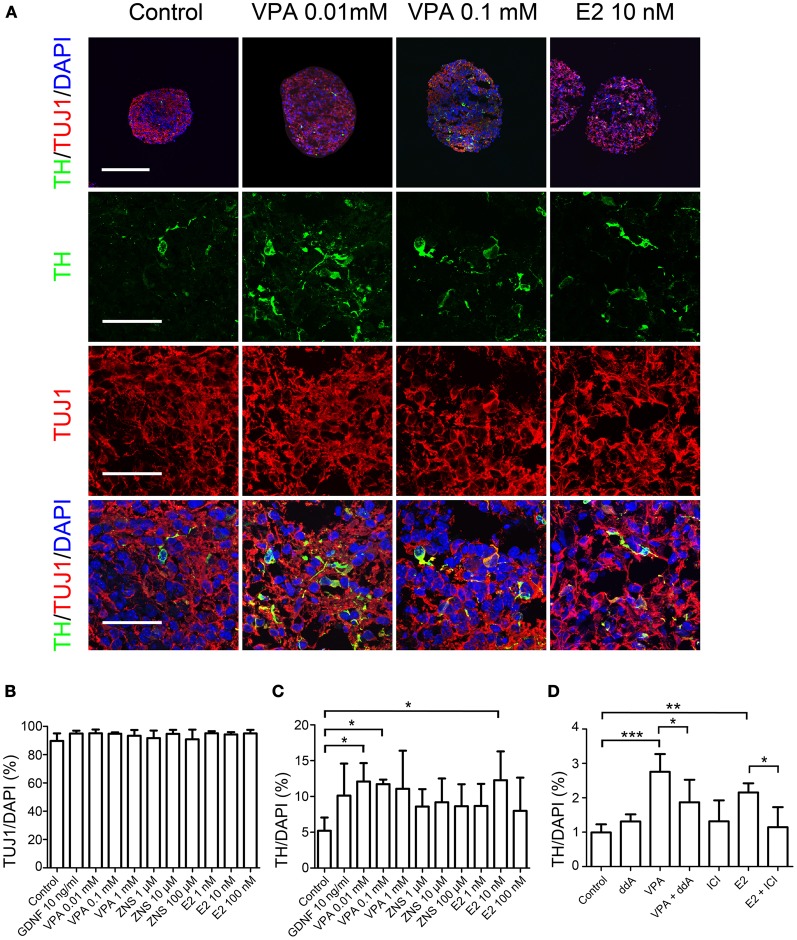
**Differentiation of iPSCs under VPA, ZNS, and E2 treatment *in vitro*.** Suspension cultures of murine iPSCs (440A3) were selected for PSA-NCAM^+^ neural progenitor cells on day 9, re-aggregated and then treated for 4 days with GDNF (as a positive control), VPA, ZNS, and E2. On day 14, the aggregates were fixed, sliced into 10-μm-thick sections and subjected to an immunocytochemical analysis for the expressions of neuronal (Tuj1) and dopaminergic (TH) markers. In the experiments for inhibition, ddA (100 μM) or ICI (2 μM) was added to the culture medium between days 10 and 14 either alone or in combination with VPA (0.1 mM) or E2 (10 nM), respectively. **(A)** Representative immunofluorescent images for TH/Tuj1 in the control and drug-treated cultures. The scale bar applies to all pictures and represents 200 μm (top) and 50 μm (lower). **(B–D)** A comparison of the number of cells labeled for Tuj1 **(B)** and TH **(C,D)** per the total number of cells. The data are presented as the mean ± SD. (^*^P < 0.05, ^**^P < 0.01, ^***^P < 0.001; *n* = 3).

First, we examined whether the test drugs, VPA, ZNS, and E2, affected the differentiation of DA neurons *in vitro*. In addition to these three drugs, GDNF was used as a positive control since it has been shown to support DA neurons *in vitro* (Young et al., 2010) and *in vivo* (Sinclair et al., 1996; Yurek, 1998). Re-aggregated PSA-NCAM^+^ cells were treated with these drugs for 4 days starting on day 10. Immunocytochemistry performed on day 14 revealed that more than 90% of the cells expressed the neuronal marker Tuj1 under all conditions (Figures 2A,B) and that 5.2 ± 1.1% of the control cells were TH^+^ (Figures 2A,C). The percentage of TH^+^ neurons significantly increased, by ~2-fold, when the cells were treated with 0.01 mM or 0.1 mM VPA, or 10 nM E2 (12.1 ± 1.5, 11.7 ± 0.4, or 12.2 ± 2.3%, respectively). To investigate whether this effect of VPA and E2 is mediated through the cyclic AMP pathway or the estrogen receptor, we used an adenylate cyclase inhibitor ddA (DeCastro et al., 2005), or an estrogen receptor antagonist ICI, respectively. When 100 μM of ddA or 2 μM of ICI were added simultaneously with 0.1 mM of VPA or 10 nM of E2, respectively, for 4 days, the increase in TH^+^ neurons was reduced significantly (Figure 2D). Addition of ddA or ICI alone did not change the percentage of TH^+^ neurons.

**Figure 3:**
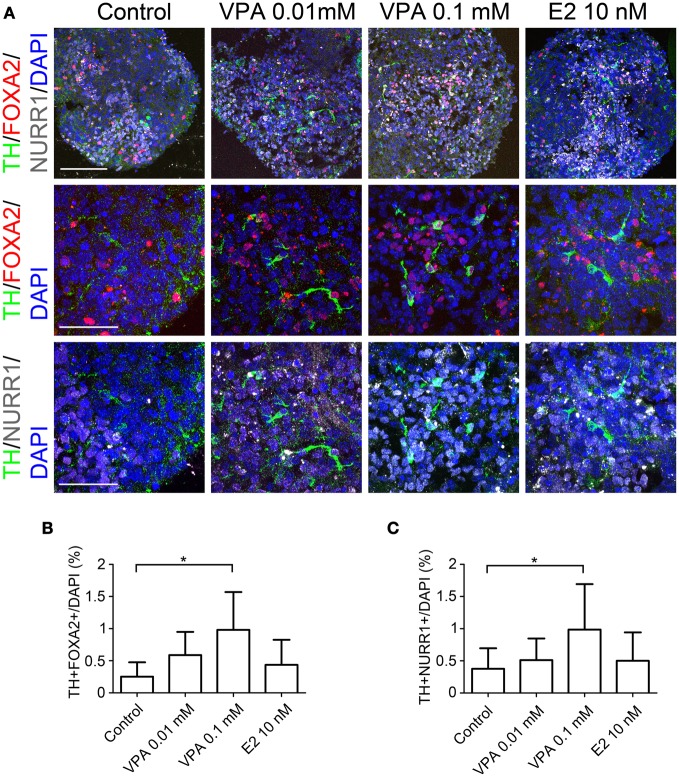
**Generation of midbrain DA neurons from iPSCs under VPA and E2 treatment *in vitro*.** On day 14, the aggregates subjected to an immunocytochemical analysis for the expressions of midbrain DA neuron markers. **(A)** Representative immunofluorescent images of TH, FOXA2, and NURR1 in the control and drug-treated cultures. The scale bar applies to all pictures and represents 100 μm (top) and 50 μm (lower). **(B,C)** A comparison of the number of cells labeled for both TH and FOXA2 **(B)** or TH and NURR1 **(C)** per the total number of cells. The data are presented as the mean ± SD. (^*^P < 0.05; *n* = 6).

In order to further characterize the TH^+^ neurons, we performed double-labeled immunocytochemistry for the markers of midbrain DA neurons, including FOXA2, NURR1, and PITX3 alongside TH. The percentages of TH^+^ FOXA2^+^, and TH^+^NURR1^+^ cells significantly increased when the cells were treated with 0.1 mM of VPA compared to that observed under the control conditions (1.00 ± 0.58% vs. 0.25 ± 0.22%, and 1.00 ± 0.70% vs. 0.37 ± 0.32%, respectively; Figure 3). There were only a few PITX3^+^ cells (<0.1% of the total number of cells), most likely because the period for differentiation was too short and there were no supportive cytokines such as GDNF in the culture medium. These results suggest that VPA treatment promotes DA differentiation and the acquisition of a midbrain-like DA neuron phenotype.

**Figure 4:**
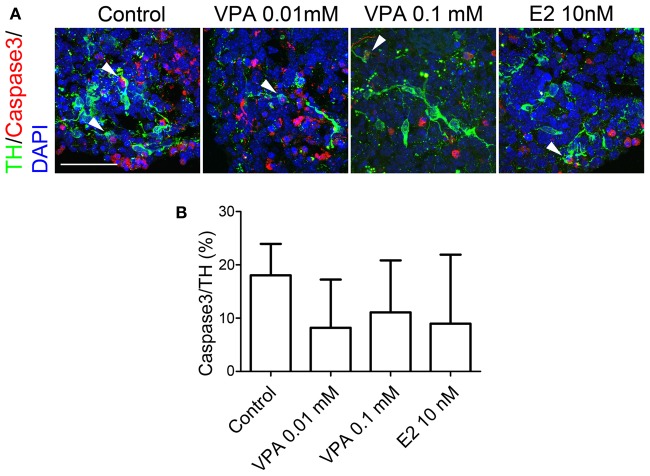
**Apoptosis of iPSC-derived TH^+^ cells under VPA, ZNS, and E2 treatment *in vitro*.** On day 14, the aggregates were subjected to an immunocytochemical analysis for dopaminergic (TH) markers as well as caspase 3, which is expressed by apoptotic cells. **(A)** Representative immunofluorescent images of TH (green) and caspase 3 (red) in the control and drug-treated cultures. The arrowheads indicate double-positive cells. The scale bar applies to all pictures and represents 50 μm. **(B)** A comparison of the number of TH^+^ cells labeled for caspase 3. The data are presented as the mean ± SD (no significant differences; *n* = 6).

Next, we evaluated whether the drugs affected the survival of TH^+^ neurons by labeling apoptotic cells for caspase 3 expression. In control spheres, 18.0 ± 5.9% of TH^+^ neurons were also labeled with caspase 3, indicating that 1/5 of the DA neurons were undergoing apoptosis (Figure 4). Figure 4B shows that VPA- (8.2 ± 9.0% by 0.01 mM, 11.1 ± 9.7% by 0.1 mM) and E2- (8.9 ± 12.9% by 10 nM)) treatment tended to yield lower percentages of apoptotic DA neurons; however, there were no significant differences in the percentages of apoptotic fractions of DA neurons between the four groups.

### VPA promoted neuronal differentiation of grafted NPCs

Next, we investigated whether the systemic administration of VPA, ZNS, or E2 influenced the survival and differentiation of DA neurons in the grafts. In this transplantation study, unsorted cell populations (3.1 × 10^5^ cells in two aggregates, in PBS) were injected into the striata of intact SD rats on day 9. The rats received intraperitoneal injections of one of the drugs and the immunosuppressant CsA every day starting 2 days before the transplantation and continuing until the day of sacrifice at 4 weeks. On the day of sacrifice, the blood concentration of CsA was 3700 ± 898 ng/ml on average. The blood concentrations of VPA, ZNS, and E2 were 158.5 ± 3.9 μg/ml, 2.43 ± 0.13 μg/ml, and 1141 ± 926 pg/ml, respectively.

**Figure 5:**
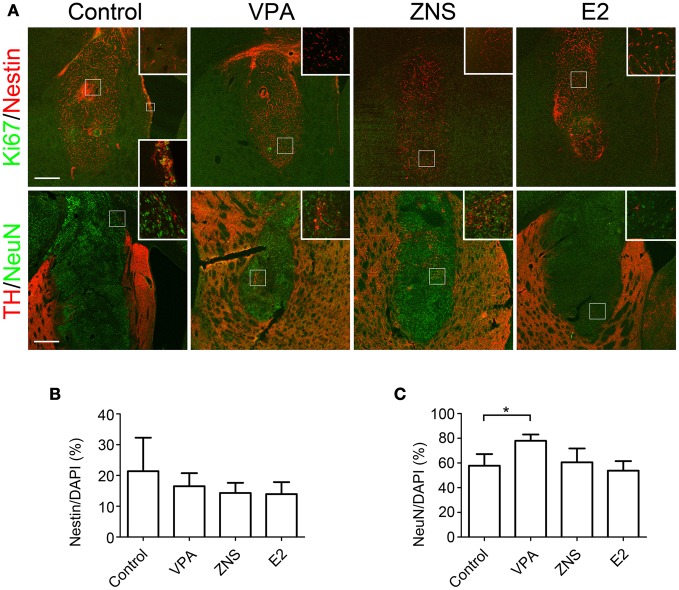
**Analyses of iPSC-derived transplants in animals treated with VPA, ZNS, or E2.** On day 9, aggregations of differentiated cells were injected into the striata of intact SD rats (3.1 × 10^5^ cells in two aggregates/tract). The rats received intraperitoneal injections of saline, VPA, ZNS, or E2. **(A)** Representative immunohistologic images of nestin (immature NPCs; red) and Ki67 (proliferation marker; green), post-mitotic neurons (NeuN; green), and DA neurons (TH; red). A magnified image of the boxed area is shown in the inlet. The lower inlet displays a high magnification of the proliferating neural precursors lining the ventricular surface as a positive control for Ki67 staining. The scale bar applies to all pictures and represents 500 μm. A comparison between each group for **(B)** the number of Nestin^+^ cells per the total number of cells in the graft, **(C)** the number of NeuN^+^ cells per the total number of cells in the graft. The data are presented as the mean ± SD. (^*^P < 0.05; *n* = 8 control, *n* = 6 VPA, ZNS, E2).

Double-labeled immunohistochemistry at 4 weeks post-transplantation against Nestin (an NPC marker) and Ki67 (a marker of proliferating cells) revealed that 15–20% of the grafted cells were Nestin^+^, but again there were no statistically significant differences (Figures 5A,B). The percentages of Ki67^+^ cells per Nestin^+^ cells were quite low in all grafts (<0.1%), suggesting that the Nestin^+^ cells were mostly quiescent or becoming post-mitotic at that time point. In contrast, immunohistochemistry against NeuN, a mature neuronal marker, revealed that the percentage of NeuN^+^ cells per the total number of cells was significantly increased when the animals were treated with VPA (77.9 ± 5.1% vs. 57.7 ± 9.4% in the control group; Figures 5A,C). These results suggest that VPA promoted the neuronal differentiation of the grafted NPCs.

**Figure 6:**
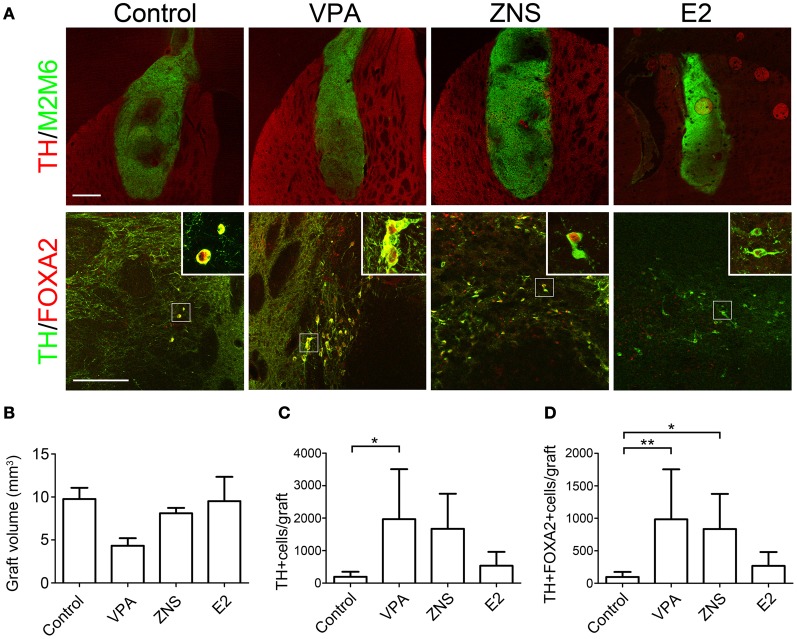
**Analyses of iPSC-derived midbrain DA neurons in animals treated with VPA, ZNS, or E2. (A)** Representative immunohistologic images of grafts (M2M6; green) containing DA neurons (TH; red), FOXA2 (ventral-midbrain marker; red), and TH (DA neuron marker; green). The scale bar applies to all pictures and represents 500 μm (upper) and 50 μm (lower). A comparison between each group for **(B)** average graft volume, **(C)** the number of TH^+^ cells per graft, and **(D)** the number of TH^+^FOXA2^+^ cells (midbrain DA neurons) per graft. The data are presented as the mean ± SD. (^*^P < 0.05, ^**^P < 0.01; *n* = 8 control, *n* = 6 VPA, ZNS, E2).

The grafted cells were identified using immunofluorescent staining for M2M6, which is only expressed by mouse cells and not by the host rat cells. At 4 weeks post-transplantation, labeling with M2M6 revealed that the grafts in all groups survived well, with no signs of tumor formation (Figure 6A, *n* = 8 in the control group, *n* = 6 in the VPA, ZNS, and E2 groups). The average estimated graft volume was smallest in the VPA-treated animals (4.33 ± 2.14 mm^3^) and was as large as 9.76 ± 3.19 mm^3^ in the control group, but there were no statistically significant differences (Figure 6B).

### Enhanced survival of midbrain DA neurons in VPA- and ZNS-treated grafts

Based on our *in vitro* data showing that the test drugs increased the number of TH^+^ neurons, we compared the number of TH^+^ neurons in the grafts of each group at 4 weeks post-transplantation. A double-labeled immunofluorescence study revealed that the VPA-treated grafts yielded significantly higher numbers of TH^+^ cells than the control grafts (1396 ± 864 and 393 ± 311, respectively; Figures 6A,C). Only a small fraction of total TH^+^ neurons co-expressed the midbrain marker FOXA2 in the control grafts (24.7 ± 9.3%). In contrast, the majority of TH^+^ neurons in the VPA- and ZNS-treated grafts were FOXA2^+^ (81.8 ± 33.6% and 80.4 ± 21.1%, respectively). The statistical analysis demonstrated that the number of midbrain DA neurons (TH^+^FOXA2^+^) in the grafts were significantly increased in the animals treated with VPA or ZNS compared to those observed in the controls (984 ± 770, 835 ± 540, and 97 ± 76, respectively; Figures 6A,D). Overall, these observations indicate that the systemic administration of VPA and ZNS increased the yield of midbrain DA neurons *in vivo* by promoting the DA differentiation of the grafted NPCs.

## Discussion

In this study, we investigated whether VPA, ZNS, or E2 affects the survival and differentiation of DA neurons derived from murine iPSCs. We found that treatment with VPA at 0.1 mM significantly increased the percentage of TH^+^FOXA2^+^ and TH^+^NURR1^+^ DA neurons derived from the iPSC-derived NPCs *in vitro*. In the subsequent transplantation study, the systemic administration of VPA and ZNS significantly improved the yield of TH^+^FOXA2^+^ DA neurons in the grafts.

Here, for the first time, we showed that a low dose of VPA (0.01 or 0.1 mM) is advantageous in promoting DA differentiation from murine iPSC-derived NPCs *in vitro*. The positive effects of VPA on neuronal differentiation have been demonstrated in a previous study (Hsieh et al., 2004); however, the effects on the differentiation of DA neurons have not been well-documented. In a study by DeCastro *et al.*, a short chain fatty acid including VPA was shown to induce TH mRNA transcription in the pheochromocytoma cell line PC12 through the cyclic AMP-dependent signaling pathway (DeCastro et al., 2005). Our result that simultaneous addition of an adenylate cyclase inhibitor ddA with 0.1 mM of VPA suppressed the effects of VPA suggests that the increased DA differentiation induced by VPA is mediated by the cyclic AMP-dependent pathway. On the other hand, 1 mM of VPA, a moderate dose that corresponds to the therapeutic plasma concentration (Blaheta and Cinatl, 2002), seemed to increase the rate of apoptosis of DA neurons. This observation corroborates the study by Hsieh in which VPA increased the apoptosis as well as the neuronal differentiation of NPCs in a dose-dependent manner (Hsieh et al., 2004). Due to the fragile nature of differentiated DA neurons, the cytotoxic effect of 1 mM of VPA might have been more prominent than the TH-inducing effect. VPA is also known to be a histone deacetylase (HDAC) inhibitor, associated with the increased transcription of a variety of factors that may contribute to the protection of midbrain DA neurons, including free radical scavengers, heat-shock proteins, and anti-apoptotic bcl-2 family members (Kidd and Schneider, 2010, 2011). Therefore, the potential mechanisms of VPA may be multiple and remain unclear. Extensive exploration is needed.

Despite the lack of noteworthy effects *in vitro*, ZNS administration resulted in an increase in the yield of TH^+^FOXA2^+^ DA neurons in the grafts. A recent study showed that ZNS supported DA neurons *in vitro* via an indirect effect mediated by astrocytes (Asanuma et al., 2010). Asanuma et al. demonstrated that ZNS markedly increases glutathione, a powerful anti-oxidant, in astrocytes and acts as a neuroprotectant against oxidative stress in 6-OHDA-lesioned rats. Therefore, the absence of astrocytes in the culture (>95% were TUJ1^+^ neurons) likely hindered ZNS from exerting such beneficial effects *in vitro*. On the other hand, the *in vivo* effects of ZNS were likely mediated by astrocytes that are abundant in the host brain. The neuroprotective effects of ZNS in PD model animals and the effects in enhancing TH have been reported in several other studies (Yano et al., 2009; Sonsalla et al., 2010; Choudhury et al., 2011). Therefore, we suggest that the systemic administration of ZNS can also be an effective strategy for increasing the yield of TH^+^FOXA2^+^ DA neurons obtained from grafted NPCs.

In addition to VPA at a concentration of 0.01 or 0.1 mM, treatment with E2 at a concentration of 10 nM also resulted in a good yield of TH^+^ neurons *in vitro*. These effects of increasing the number of DA neurons *in vitro* are consistent with the previous findings of our group and others in which E2 increased the proliferation of DA progenitors through actions mediated by estrogen receptors (Kishi et al., 2005; Díaz et al., 2009). Increased proliferation induced by E2 has also been observed more generally in the NSCs of rats (Brännvall et al., 2002) and humans (Wang et al., 2008). Despite the favorable results obtained *in vitro*, the systemic administration of E2 resulted in a poor yield of DA neurons in the transplants. We might have observed more TH^+^ cells in the grafts if there was a more appropriate concentration for E2 administration or if we had examined the samples at a later time point rather than at 4 weeks post-transplantation.

Approximately 20–75% of the TH^+^ cells in the grafts did not express FOXA2, suggesting that they are not of the midbrain but possibly of the forebrain subtype of DA neurons. In addition, ~95% of the NeuN^+^ cells in the grafts did not express TH, indicating that they are not DA but rather another type of neuron. Although the protocol used to generate midbrain DA neurons from iPSCs has been developing, the one used in the present study is not yet perfect. To achieve more abundant survival of midbrain DA neurons, further improvements in the protocol and purification of midbrain DA progenitor cells are needed. We attempted to transplant PSA-NCAM^+^ cells after FACS, which resulted in a low survival rate of the grafted cells, particularly DA neurons (less than 50 TH^+^ cells per graft under each condition). Therefore, the method of cell sorting and recovery after sorting also needs to be improved.

In conclusion, we herein demonstrated improved survival and differentiation of iPSC-derived DA neurons showing a midbrain-like phenotype following the systemic administration of well-established anti-epileptic drugs, VPA, and ZNS. Recent efforts in the field of stem cell therapy have primarily focused on deriving more genuine cells of clinically relevant cell types such as midbrain DA neurons from pluripotent stem cells. It is, however, equally important to develop strategies to modify the microenvironment of the host brain in order to achieve the optimal results of such therapies. Because there are so many safety issues to overcome before pluripotent stem cells can be used for the treatment of otherwise incurable diseases, we believe that taking advantage of drugs that are already safely used can save valuable time in the clinical application of stem cell technology.

### Conflict of interest statement

The authors declare that the research was conducted in the absence of any commercial or financial relationships that could be construed as a potential conflict of interest.
