# Erratum: Systemic administration of valproic acid and zonisamide promotes differentiation of induced pluripotent stem cell-derived dopaminergic neurons

**DOI:** 10.3389/fncel.2013.00116

**Published:** 2013-07-22

**Authors:** Tatsuya Yoshikawa, Bumpei Samata, Aya Ogura, Susumu Miyamoto, Jun Takahashi

**Affiliations:** ^1^Institute for Frontier Medical Sciences, Kyoto UniversityKyoto, Japan; ^2^Department of Neurosurgery, Mie University Graduate School of MedicineTsu, Japan; ^3^Department of Neurosurgery, Kyoto University Graduate School of MedicineKyoto, Japan; ^4^Center for iPS Cell Research and Application, Kyoto UniversityKyoto, Japan

The Figure 6C in the article is corrected.

**Figure 6:**
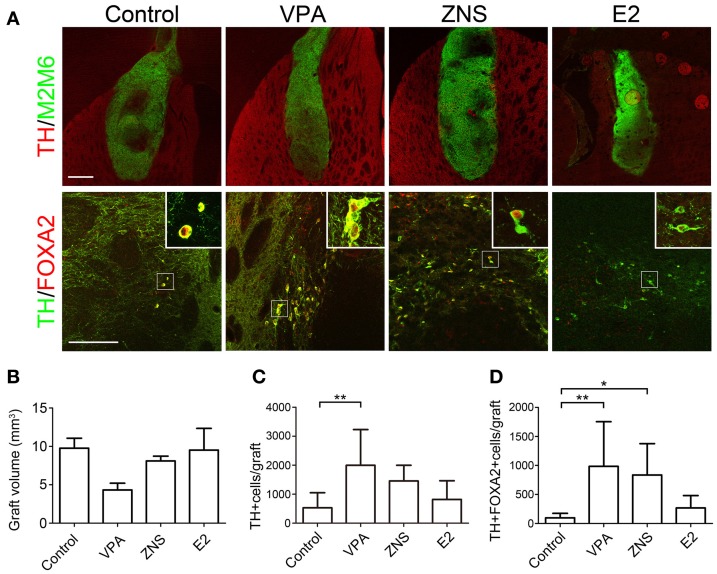
**Analyses of iPSC-derived midbrain DA neurons in animals treated withVPA, ZNS, or E2. (A)** Representative immunohistologic images of grafts (M2M6; green) containing DA neurons (TH; red), FOXA2 (ventral-midbrain marker; red), and TH (DA neuron marker; green). The scale bar applies to all pictures and represents 500 μm (upper) and 50μm (lower). A comparison between each group for **(B)** average graft volume, **(C)** the number of TH+ cells per graft, and **(D)** the number of TH+FOXA2+ cells(midbrain DA neurons) per graft. The data are presented as the mean ± *SD*. (^*^P < 0.05, ^**^P < 0.01; *n* = 8 control, *n* = 6 VPA, ZNS, E2).

